# *Plasmodium* species positivity rates across regions with different malaria transmission intensities in Mainland Tanzania: Data from a nationwide survey conducted in 2023

**DOI:** 10.1371/journal.pgph.0006091

**Published:** 2026-11-12

**Authors:** Salehe S. Mandai, Catherine Bakari, Daniel A. Petro, Angelina J. Kisambale, Gervas A. Chacha, Dativa Pereus, Hussein J. Semboja, Rashid A. Madebe, Daniel Mbwambo, Sijenunu Aaron, Abdallah Lusasi, Samwel Lazaro, Celine I. Mandara, Misago D. Seth, Deus S. Ishengoma

**Affiliations:** 1 Ifakara Health Institute, Dar es Salaam, Tanzania; 2 National Institute for Medical Research, Dar es Salaam, Tanzania; 3 University of Dar es Salaam, Dar es Salaam, Tanzania; 4 Muhimbili University of Health and Allied Sciences, Dar es Salaam, Tanzania; 5 National Malaria Control Programme, Dodoma, Tanzania; University of Cape Town, SOUTH AFRICA

## Abstract

Although *Plasmodium falciparum* is the most prevalent and highly virulent species in Sub-Saharan Africa, non-falciparum species are increasingly recognized as a hidden burden. Generally, infections caused by non-falciparum species occur with low parasite densities and often present as co-infections with *P. falciparum*. These parasites may evade detection by routine diagnostics, complicating malaria case management and control efforts. To investigate the distribution of different *Plasmodium* parasites, we analysed 5,314 randomly selected samples from 18,969 samples collected across all 26 regions of Mainland Tanzania in 2023. *Plasmodium* parasites were detected using 18S qPCR. Of the analyzed samples, 52.3% (n = 2,780) were positive for any *Plasmodium* species. *P. falciparum* was detected in 51.2% (2,719/5,314), *P. malariae* in 1.4% (76/5,314), and *P. ovale* spp. in 1.8% (98/5,314) of the samples. Mixed infections were detected in 2.0% (107/5,314), with *P. malariae* and *P. falciparum* in 0.8% (40/5,314), and *P. ovale* mixed with *P. falciparum* in 1.1% (61/5,314) of the samples analysed. Among the samples with positive results (n = 2,780), mono-infections were detected at 93.9% (2,612/2,780) for *P. falciparum*, 1.1% (30/2,780) for *P. malariae*, and 1.1% (31/2,780) for *P. ovale* spp., respectively. Unlike *P. malariae* and *P. ovale*, *P. falciparum* infections were significantly influenced by transmission intensities, with the highest odds in regions with high transmission intensities compared to those with low (OR=1.61, *p* < 0.001), and very low transmission (OR=1.48, *p* < 0.001). School children had higher odds of *P. falciparum* infections compared to adults (OR=1.56, *p* < 0.001) and under-fives (OR=1.47, *p* < 0.001). Adults were also less likely to have *P. malariae* infections than schoolchildren (OR= 0.45, *p* = 0.021).The findings of the first-ever nation-wide survey reveal a high prevalence of *P. falciparum*, low prevalence of non-falciparum species, and complex species landscape. They underpin the need to expand surveillance systems beyond *P. falciparum* and adapt interventions to target all *Plasmodium* species.

## Introduction

Malaria remains one of the most important public health concerns, particularly in Africa, which accounted for 265 million malaria cases and 579,000 deaths, representing 94% of global cases and 95% of deaths in 2024 [1]. Malaria is particularly prevalent and deadly in Sub-Saharan Africa (SSA), especially in malaria-endemic areas [2]. In 2024, five SSA countries, including Nigeria (24.3%), the Democratic Republic of the Congo (12.5%), Uganda (4.7%), Ethiopia (4.4%), and Mozambique (3.6%), accounted for over half of global malaria cases [1]. Likewise, Nigeria (30.3%), the Democratic Republic of the Congo (11.1%), Niger (5.8%), and the United Republic of Tanzania (4.3%), all from SSA, contributed to more than half of global malaria deaths in the same year [1]. To address this persistent burden, intensified prevention, diagnosis, treatment, and surveillance efforts are essential, particularly in the high-burden regions of SSA.

Malaria is a mosquito-borne disease caused by *Plasmodium* parasites, primarily *Plasmodium falciparum*, *Plasmodium vivax*, *Plasmodium malariae*, *Plasmodium ovale*, and *Plasmodium knowlesi* [3,4]. Among these, *P. falciparum* and *P. vivax* are the deadliest human malaria species due to their high morbidity and mortality rates, ability to cause severe complications, widespread drug resistance, and unique biological adaptations that enable persistence and rapid disease progression [5,6]. *P. falciparum* is Africa's most virulent and dominant species, responsible for over 80% of malaria-related deaths in SSA [7,8]*.* Its ability to cause severe manifestations and multiple organ failure makes it a major public health concern [9,10]. Moreover, *P. falciparum* has developed resistance to multiple antimalarial drugs, including chloroquine and sulfadoxine/pyrimethamine, and mostly recently artemisinins [11–14], further complicating treatment and control efforts. In contrast, *P. vivax* is more prevalent in Latin America and Southeast Asia [15,16] and the most widespread outside Africa [15,17]. While *P. vivax* causes less severe malaria than *P. falciparum,* its ability to relapse from dormant liver stages (hypnozoites), reticulocyte tropism, and rapid gametocyte development contribute to recurrent infections and sustained transmission, making it more challenging to control [18,19]. Recent studies reported that *P. vivax* has shown increasing evidence of drug resistance, which could complicate its management if not closely monitored [20,21].

Although less prevalent*, P. malariae*, *P. ovale*, and *P. knowlesi* present additional challenges for malaria control [22]. *P. malariae* is known to cause a chronic, mild infection, which can persist for years if untreated [23]. Its asymptomatic nature often leads to underdiagnosis, serving as a reservoir for transmission and potentially contributing to chronic anaemia and co-infections, further complicating efforts to reduce transmission [24]. Similarly, *P. ovale*, which primarily occurs in tropical Africa and parts of Asia but is especially prevalent in West Africa [25], can cause relapsing malaria due to its ability to form hypnozoites in the liver, though it is generally considered less virulent than *P. falciparum* or *P. vivax* [26,27]. However, its ability to relapse and go undetected in routine surveillance can hinder control efforts, especially in areas with limited diagnostic resources. *P. knowlesi*, a zoonotic species primarily found in Southeast Asia [28,29], has gained attention due to its ability to cause severe malaria in humans, often leading to severe and even fatal malaria complications if not promptly treated [30–32]. The rising number of infections linked to *P. knowlesi* underscores the need for heightened surveillance and awareness of *non-falciparum* species.

For many years, non-falciparum *Plasmodium* species (*P. vivax, P. malariae,* and *P. ovale* spp.) have been considered relatively benign and less prevalent in Africa [33,34]. However, evidence has emerged that these species are more prevalent than previously thought [35,36]. Most of these species typically co-infect with *P. falciparum* and present with low parasitaemia, making them more likely to be missed with the currently used rapid diagnostic tests [37–39]. This under-reporting presents a significant challenge, as co-infections with less prevalent species can complicate diagnosis and treatment, leading to suboptimal clinical management. Current case management strategies, which are primarily focused on the more dominant *P. falciparum*, may not be sufficient to curb the effects of other *Plasmodium* species, especially when they present with low parasitaemia [40]. The failure to detect these species may allow them to persist and contribute to the overall malaria burden. To effectively combat malaria, it is essential to enhance diagnostic capabilities and adapt case management strategies to account for the full spectrum of *Plasmodium* species, ensuring more comprehensive and accurate treatment. Without addressing these overlooked species, current malaria control efforts may fall short in reducing the disease burden, particularly in regions where co-infections are common.

In Tanzania, malaria is still a significant public health challenge, and the country is experiencing heterogeneous transmission [41–43]. Despite sustained control efforts, variations in transmission intensity between regions continue to complicate elimination goals, suggesting that existing surveillance and intervention approaches may not fully capture the evolving malaria landscape, which could have important implications for diagnosis and control. Current diagnosis and treatment efforts have primarily focused on falciparum malaria, with a passive surveillance system that relies heavily on rapid diagnostic tests, which are less sensitive at detecting other *Plasmodium* species. This falciparum-centric approach underestimates the contributions of non-falciparum species to the overall malaria burden, thereby limiting the effectiveness of case management and control strategies. Although previous studies have confirmed the presence of non-falciparum *Plasmodium* species in Tanzania [40,44], their epidemiology remains poorly understood due to limited geographic scope and small sample sizes. Consequently, significant data gaps persist across much of the country. To address this, the present study analysed the largest dataset to date, spanning all 26 regions of Mainland Tanzania, to provide a comprehensive assessment of *Plasmodium* species across diverse transmission settings.

## Methods

### Study sites and population

This study utilised data and samples from a health facility-based cross-sectional study that was conducted between January 23 and August 28, 2023, in all 26 regions of Mainland Tanzania. The survey was conducted under the project on molecular surveillance of malaria in Mainland Tanzania (MSMT), which primarily focused on malaria parasite population genetics, drug resistance markers, and surveillance of histidine-rich protein 2/3 (*hrp2/3*) gene deletions as previously described [13,45]. The project was initially implemented in 13 regions with different levels of malaria transmission intensities as previously described [45]. From January 2023, the MSMT project was extended to cover all 26 regions of Mainland Tanzania to provide important information to National Malaria Control Programme (NMCP) from the regions that were not covered in 2021 and 2022. For the 2023 survey, 86 health facilities (HFs) from all 26 regions were selected for inclusion in this study, with a target of at least three facilities per region as previously described [46]. Based on the 2020 malaria stratification by the NMCP, the selected sites included 28 HFs in high, 31 in moderate, 12 in low, and 15 in very low transmission strata, as shown in Fig 1.

**Fig 1:**
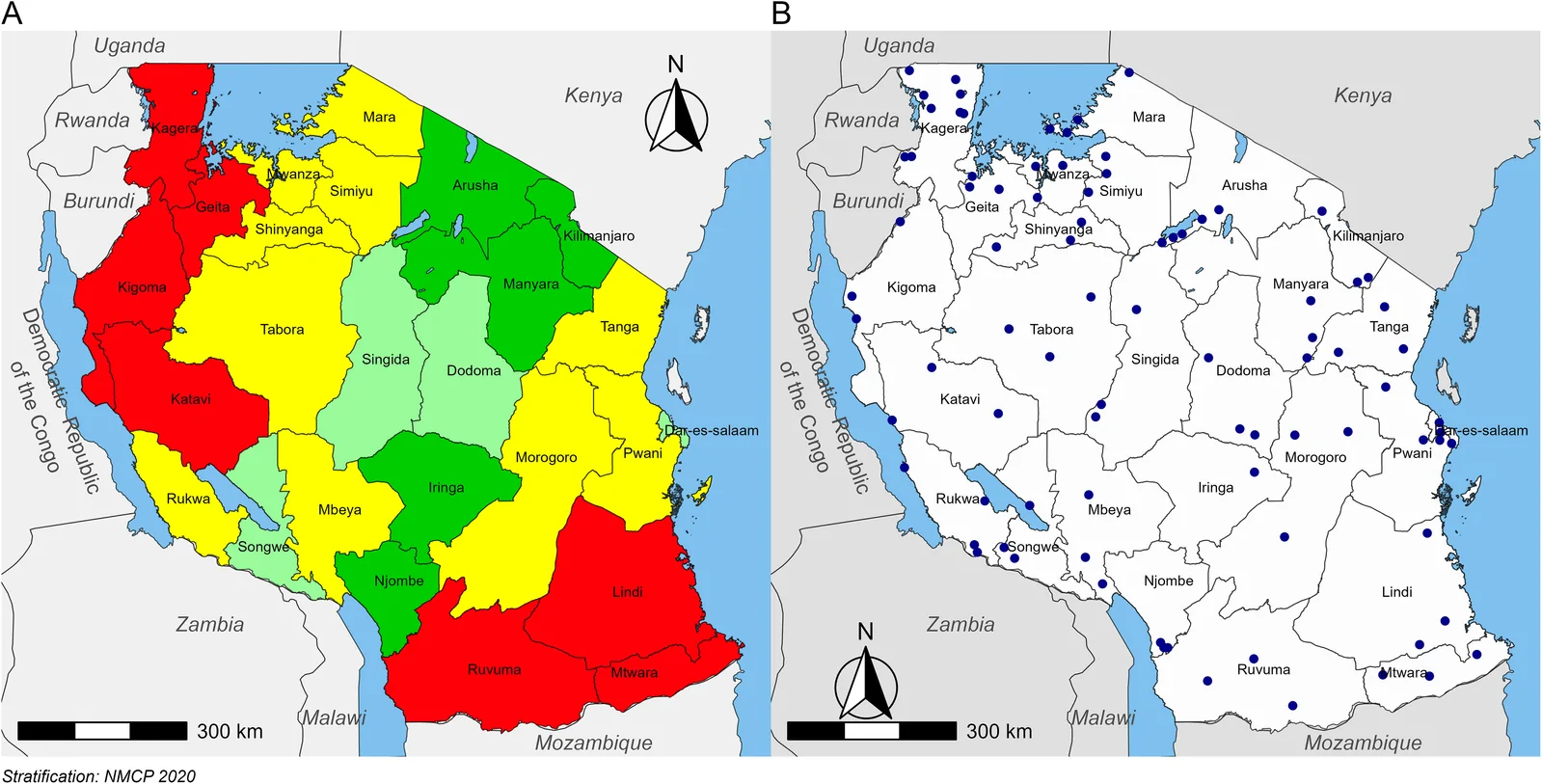
Maps of Tanzania showing malaria stratification and study sites; *(A)* Malaria stratification based on classifications from the NMCP, depicting variations in malaria endemicity across the country. Colours indicate transmission strata: high (red), moderate (yellow), low (light green), and very low (green). ***(B)*** Study sites where data collection was conducted. Administrative boundaries were obtained from GADM (https://gadm.org/; license: https://gadm.org/license.html).

### Sample collection

Briefly, the survey recruited febrile outpatients aged ≥6 months who presented at the HFs with suspected uncomplicated malaria and resided within the same catchment area as the respective HF [40]. Individual informed consent was sought as previously described by Rogier *et al* [45]. For each participant, finger-prick blood was collected for malaria parasite detection by RDTs and preparation of dried blood spots (DBS) on Whatman 3 MM filter papers (Cytiva, Marlborough, MA, USA) as described previously [45]. In all 86 HFs, 18,969 DBS samples were collected and stored in zipper bags containing silica gel. These samples were kept at ambient temperature at the respective HFs and later transported to the National Institute for Medical Research (NIMR) Genomics Laboratory after the survey for further analysis. Out of 18,969 samples collected, a random subset of 5,314 samples was selected for molecular analysis to determine the distribution of the different *Plasmodium* species across the country. Sampling was performed proportionally according to the number of samples collected within each region to ensure representation across all study settings.

### Molecular speciation assays

For each sample, three 6-*mm* DBS punches were obtained and placed into deep well plates. DNA extraction was done using the Tween-Chelex 100 (Bio-Rad Laboratories, USA) extraction method as previously described, with some modifications [47]. Quantitative real-time PCR (qPCR) assays targeting the 18S ribosomal subunit were performed using the Bio-Rad CFX 96 Opus real-time PCR detection system (Bio-Rad, California, USA), with CFX Maestro software version 2.2 as previously described [48,49]. Separate qPCR assays were conducted for *P. falciparum, P. malariae,* and *P. ovale*. The *P. ovale* assay did not distinguish between the two *P. ovale* subspecies (*P. ovale curtisi* and *P. ovale wallikeri*). The *P. malariae* assay used 42 cycles, whereas the remaining assays for *P. falciparum* and *P. ovale* used 45 cycles. Samples were considered positive when amplification showed the sigmoid curve with Ct value < 40. Semiquantitative parasitaemia was determined using standard curves generated from a 10-fold serial dilution of plasmids, as previously described [48,49]. *P. vivax* analysis was not performed due to its rare detection in recent studies [40,44].

### Statistical analysis

Descriptive statistics, including frequencies, percentages, means, and interquartile ranges (IQR), were used to summarize the data. Pearson’s Chi-Square test was employed to assess differences in positivity rates of *Plasmodium species* across age groups of under-fives (<5 years), school children (5– < 15 years), and adults (≥15 years), and strata (high, moderate, low, and very low). Fisher’s Exact Test was employed for contingency tables with small, expected frequencies (<5). Post hoc pairwise comparisons were conducted using a Bonferroni correction to identify specific group differences. Generalised linear models (GLM) were fitted to evaluate the likelihood of positivity, and pairwise comparisons were performed using estimated marginal means (EMMs) derived from logistic regression models, reporting odds ratios (ORs) and their corresponding 95% confidence intervals, adjusted with Tukey’s method. A p-value <0.05 was considered statistically significant. Maps were created using the *sf* package (version 1.0.15) using shapefiles from gadm.org, and plots/graphs were plotted with the *tidyplots* package (version 0.2.1) and *ggplot2* package (version 3.51). All analyses were conducted in R (version 4.3.2, The R Foundation for Statistical Computing, Vienna, Austria).

### Ethics approval and consent to participate

This large health facility survey was part of the MSMT project, whose protocol was reviewed and approved by the Medical Research Coordinating Committee (MRCC) of the National Institute for Medical Research (NIMR), on 16 December 2020, with certificate number NIMR/HQ/R.8a/Vol. IX/3579. Permission to conduct the study in the different regions was sought from the President’s Office, Regional Administration and Local Government (PO-RALG). Before taking part in the survey, verbal and written informed consent was sought and obtained from all participants or parents/guardians in the case of children.

## Results

### Study population

Within this subset of 5,314 patients, the median age was 8 years (interquartile range [IQR], 21), ranging from 6 months to 93 years. Most study participants were female 58.5%; n = 3,107), while males constituted 41.5% (n = 2,207). Under-fives (<5 years old) constituted 43.8% of participants (n = 2,325), while school children (with 5- < 15 years old) were 17.5% (n = 929), and adults (≥15 years old) constituted 38.8% (n = 2,060). By transmission strata, 858 samples (16.1%) were from very low, 774 (14.6%) from low, 1,929 (36.3%) from moderate, and 1,753 (33%) from high strata. The number of samples analysed from each of the 26 regions is given in Table 1.

**Table 1:** Characteristics of study population.

Region	Total	Sex		Age group		
		Female n (%)	Male n (%)	Underfives n (%)	School children n (%)	Adults n (%)
**Very Low transmission strata**						
Arusha	189	107 (56.6)	82 (43.4)	58 (30.7)	36 (19)	95 (50.3)
Iringa	96	47 (49)	49 (51)	45 (46.9)	26 (27.1)	25 (26)
Kilimanjaro	191	103 (53.9)	88 (46.1)	25 (13.1)	26 (13.6)	140 (73.3)
Manyara	191	112 (58.6)	79 (41.4)	57 (29.8)	38 (19.9)	96 (50.3)
Njombe	191	108 (56.5)	83 (43.5)	55 (28.8)	45 (23.6)	91 (47.6)
**Subtotal**	**858**	**477**	**381**	**240**	**171**	**447**
**Low transmission strata**						
Dar es Salaam	205	98 (47.8)	107 (52.2)	63 (30.7)	23 (11.2)	119 (58)
Dodoma	194	104 (53.6)	90 (46.4)	75 (38.7)	31 (16)	88 (45.4)
Singida	190	105 (55.3)	85 (44.7)	125 (65.8)	16 (8.4)	49 (25.8)
Songwe	185	97 (52.4)	88 (47.6)	63 (34.1)	45 (24.3)	77 (41.6)
**Subtotal**	**774**	**404**	**370**	**326**	**115**	**333**
**Moderate transmission strata**						
Mara	191	107 (56)	84 (44)	46 (24.1)	66 (34.6)	79 (41.4)
Mbeya	180	106 (58.9)	74 (41.1)	77 (42.8)	25 (13.9)	78 (43.3)
Morogoro	190	134 (70.5)	56 (29.5)	74 (38.9)	36 (18.9)	80 (42.1)
Mwanza	191	112 (58.6)	79 (41.4)	91 (47.6)	47 (24.6)	53 (27.7)
Pwani	192	115 (59.9)	77 (40.1)	97 (50.5)	33 (17.2)	62 (32.3)
Rukwa	223	137 (61.4)	86 (38.6)	94 (42.2)	46 (20.6)	83 (37.2)
Simiyu	192	125 (65.1)	67 (34.9)	99 (51.6)	26 (13.5)	67 (34.9)
Shinyanga	192	108 (56.2)	84 (43.8)	106 (55.2)	40 (20.8)	46 (24)
Tabora	189	111 (58.7)	78 (41.3)	107 (56.6)	20 (10.6)	62 (32.8)
Tanga	189	116 (61.4)	73 (38.6)	46 (24.3)	55 (29.1)	88 (46.6)
**Subtotal**	**1,929**	**1,171**	**758**	**837**	**394**	**698**
**High transmission strata**						
Geita	190	114 (60)	76 (40)	106 (55.8)	32 (16.8)	52 (27.4)
Kagera	619	377 (60.9)	242 (39.1)	355 (57.4)	74 (12)	190 (30.7)
Kigoma	189	115 (60.8)	74 (39.2)	88 (46.6)	29 (15.3)	72 (38.1)
Katavi	191	129 (67.5)	62 (32.5)	58 (30.4)	34 (17.8)	99 (51.8)
Lindi	188	107 (56.9)	81 (43.1)	122 (64.9)	19 (10.1)	47 (25)
Mtwara	190	111 (58.4)	79 (41.6)	114 (60)	19 (10)	57 (30)
Ruvuma	186	102 (54.8)	84 (45.2)	79 (42.5)	42 (22.6)	65 (34.9)
**Subtotal**	**1,753**	**1,055**	**698**	**922**	**249**	**582**
**Overall**	**5314**	**3107**	**2207**	**2325**	**929**	**2060**

### qPCR positivity by species

Of these 5,314 individuals tested for all species, 47.7% (n = 2,534) showed no malaria infection by qPCR. *P. falciparum* was detected in 51.2% (n = 2,719; 95% confidence interval [CI], 49.8%–52.5%), *P. malariae* in 1.4% (n = 76; 95% CI, 1.1%–1.8%), and *P. ovale* spp. in 1.8% (n = 98; 95% CI, 1.5%–2.3%) of tested samples. Mixed-species infections were observed in 2.0% (n = 107/5,314). *P. malariae* infections were mixed with *P. falciparum* (0.8%, n = 40 of 5,314), and most *P. ovale* cases, 1.1% (61/5,314), were co-infections with *P. falciparum.* Among the positive samples (n = 2,780), mono-infections were also common, detected in 93.9% (2,612/2,780), 1.1% (30/2,780), and 1.1% (31/2,780) of *P. falciparum, P. malariae, and P. ovale* spp*.* respectively (Table 2). The triple infections with *P. malariae*, *P. falciparum,* and *P. ovale* spp were observed in 6 (0.2%/2,780) cases only. *P. malariae and P. ovale* spp mixed infections were not detected.

**Table 2:** Positivity rate by species.

Infection type	All 5,314 samples n (%)	In 2,780 positive samples only n (%)
***P. falciparum* (in all samples)**	**2,719 (51.2%)**	**2,612 (93.9)**
*P. malariae*	30 (0.6)	30 (1.1)
*P. malariae/P. falciparum*	40 (0.8)	40 (1.4)
*P. malariae/P. falciparum/P. ovale*	6 (0.1)	6 (0.2)
**All *P. malariae* infections**	**76 (1.4)**	**76 (2.7)**
*P. ovale*	31 (0.6)	31 (1.1)
*P. ovale/P. falciparum*	61 (1.1)	61 (2.2)
*P. ovale/P. falciparum/P. malariae*	6 (0.1)	6 (0.2)
**All *P. ovale* infections**	**98 (1.8)**	**98 (3.5)**

### Parasite density

The geometric mean parasite densities (GMPDs), quantified by semiquantitative qPCR, were highest for *P. falciparum* at 1,021 parasites/µL (95% CI: 903–1,155), followed by *P. malariae* at 260 parasites/µL (95% CI: 96–705) and *P. ovale* spp. at 139 parasites/µL (95% CI: 71–272). Among individuals with *P. falciparum* infections, school children exhibited significantly higher GMPDs compared to adults (β = 0.514, 95% CI: 0.378–0.651, p < 0.001), followed by under-fives (β = 0.306, 95% CI: 0.201–0.412, p < 0.001), as shown in Fig 2. For *P. malariae* infections, the GLM indicated that school children had slightly higher GMPDs than adults (β = 0.026, 95% CI: –0.004–0.057, p = 0.090); however, difference was not statistically significant. In contrast, among individuals with *P. ovale* infections, under-fives had higher GMPDs compared to adults (β = 0.025, 95% CI: 0.004–0.046, p = 0.020), while no significant difference was observed between school children and adults (β = 0.009, 95% CI: –0.013–0.032, p = 0.412).

**Fig 2:**
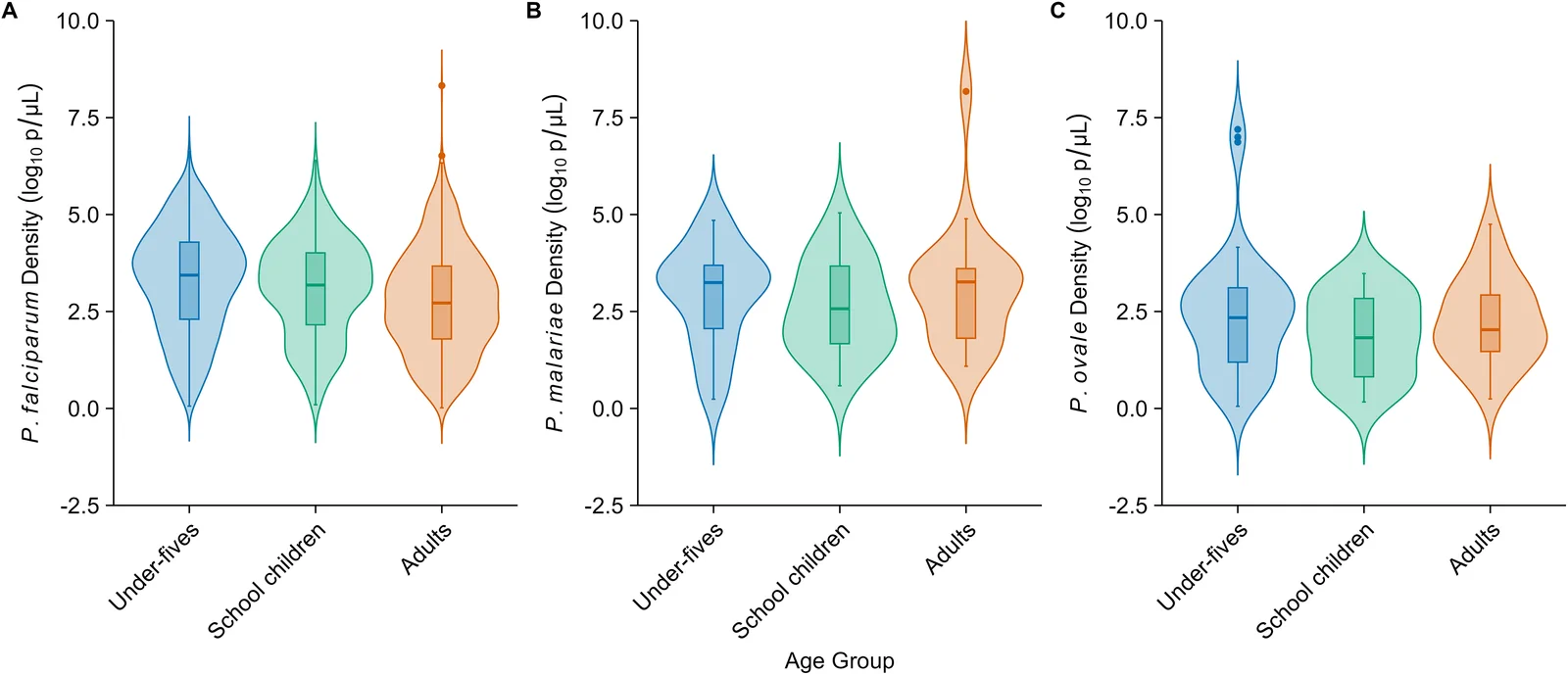
Violin plots showing the distribution of parasite densities across age groups for each *Plasmodium* species. ***(A)*** *P. falciparum,* ***(B)*** *P. malariae, and* ***(C)*** *P. ovale*.

### Variability by region and transmission strata

*P. falciparum* was detected in all sampled regions (Fig 3), with the highest positivity rates in Mtwara (67.4%, n = 128 of 190) and Njombe (66.0%, n = 126 of 191), and the lowest in Kilimanjaro (23.0%, n = 44 of 191), as shown in **Table 3**. When analysis was done based on the samples with positive results only, *P. falciparum* was still the dominant species in all regions. The highest positivity (100%) was observed in Dar es Salaam, Dodoma, Katavi, and Simiyu, while Iringa (93.3%, n = 28 of 30) and Mbeya (93.2%, n = 68 of 73) had slightly lower rates, as shown in **Table 4**. *P. malariae* was detected in several areas, with the highest positivity rates in Mara (6.8%, n = 13 of 191). At the same time, it was absent in most regions, including Arusha, Dar es Salaam, Dodoma, Lindi, Manyara, and Simiyu. *P. ovale* spp. was found in lower abundance, with notably high positivity rates in Mara (5.8%, n = 11 of 191) and Arusha (4.8%, n = 9 of 189), though it was detected at very low positivity rate in areas like Kilimanjaro, Mbeya, and Songwe and was not detected in Dodoma, Katavi, Mtwara, and Shinyanga regions (Fig 3).

**Table 3:** Positivity rates of different *Plasmodium* species in all samples analysed from the 26 regions of Mainland Tanzania.

Region	*P. falciparum*	*P. malariae*	*P. ovale*	Total Samples
Arusha	92(48.7)	0(0)	9(4.8)	189
Dar es Salaam	77(37.6)	0(0)	1(0.5)	205
Dodoma	82(42.3)	0(0)	0(0)	194
Geita	88(46.3)	7(3.7)	6(3.2)	190
Iringa	28(29.2)	2(2.1)	2(2.1)	96
Kagera	346(55.9)	17(2.7)	16(2.6)	619
Katavi	83(43.5)	1(0.5)	0(0)	191
Kigoma	102(54)	1(0.5)	3(1.6)	189
Kilimanjaro	44(23)	1(0.5)	1(0.5)	191
Lindi	115(61.2)	0(0)	6(3.2)	188
Manyara	99(51.8)	0(0)	1(0.5)	191
Mara	125(65.4)	13(6.8)	11(5.8)	191
Mbeya	68(37.8)	5(2.8)	1(0.6)	180
Morogoro	91(47.9)	3(1.6)	2(1.1)	190
Mtwara	128(67.4)	4(2.1)	0(0)	190
Mwanza	114(59.7)	2(1)	6(3.1)	191
Njombe	126(66)	1(0.5)	1(0.5)	191
Pwani	112(58.3)	1(0.5)	3(1.6)	192
Rukwa	101(45.3)	4(1.8)	6(2.7)	223
Ruvuma	105(56.5)	4(2.2)	4(2.2)	186
Shinyanga	125(65.1)	2(1)	0(0)	192
Simiyu	102(53.1)	0(0)	5(2.6)	192
Singida	98(51.6)	2(1.1)	4(2.1)	190
Songwe	78(42.2)	4(2.2)	1(0.5)	185
Tabora	103(54.5)	1(0.5)	4(2.1)	189
Tanga	87(46)	1(0.5)	5(2.6)	189

**Table 4:** Positivity rates of different *Plasmodium* species in all samples with positive results from the 26 regions of Mainland Tanzania.

Region	*P. falciparum*	*P. malariae*	*P. ovale*	Total
Arusha	92(98.9)	0(0)	9(9.7)	93
Dar es Salaam	77(100)	0(0)	1(1.3)	77
Dodoma	82(100)	0(0)	0(0)	82
Geita	88(93.6)	7(7.4)	6(6.4)	94
Iringa	28(93.3)	2(6.7)	2(6.7)	30
Kagera	346(98.9)	17(4.9)	16(4.6)	350
Katavi	83(100)	1(1.2)	0(0)	83
Kigoma	102(97.1)	1(1)	3(2.9)	105
Kilimanjaro	44(95.7)	1(2.2)	1(2.2)	46
Lindi	115(96.6)	0(0)	6(5)	119
Manyara	99(99)	0(0)	1(1)	100
Mara	125(97.7)	13(10.2)	11(8.6)	128
Mbeya	68(93.2)	5(6.8)	1(1.4)	73
Morogoro	91(95.8)	3(3.2)	2(2.1)	95
Mtwara	128(97)	4(3)	0(0)	132
Mwanza	114(97.4)	2(1.7)	6(5.1)	117
Njombe	126(99.2)	1(0.8)	1(0.8)	127
Pwani	112(98.2)	1(0.9)	3(2.6)	114
Rukwa	101(98.1)	4(3.9)	6(5.8)	103
Ruvuma	105(96.3)	4(3.7)	4(3.7)	109
Shinyanga	125(98.4)	2(1.6)	0(0)	127
Simiyu	102(100)	0(0)	5(4.9)	102
Singida	98(98)	2(2)	4(4)	100
Songwe	78(96.3)	4(4.9)	1(1.2)	81
Tabora	103(99)	1(1)	4(3.8)	104
Tanga	87(97.8)	1(1.1)	5(5.6)	89

**Fig 3:**
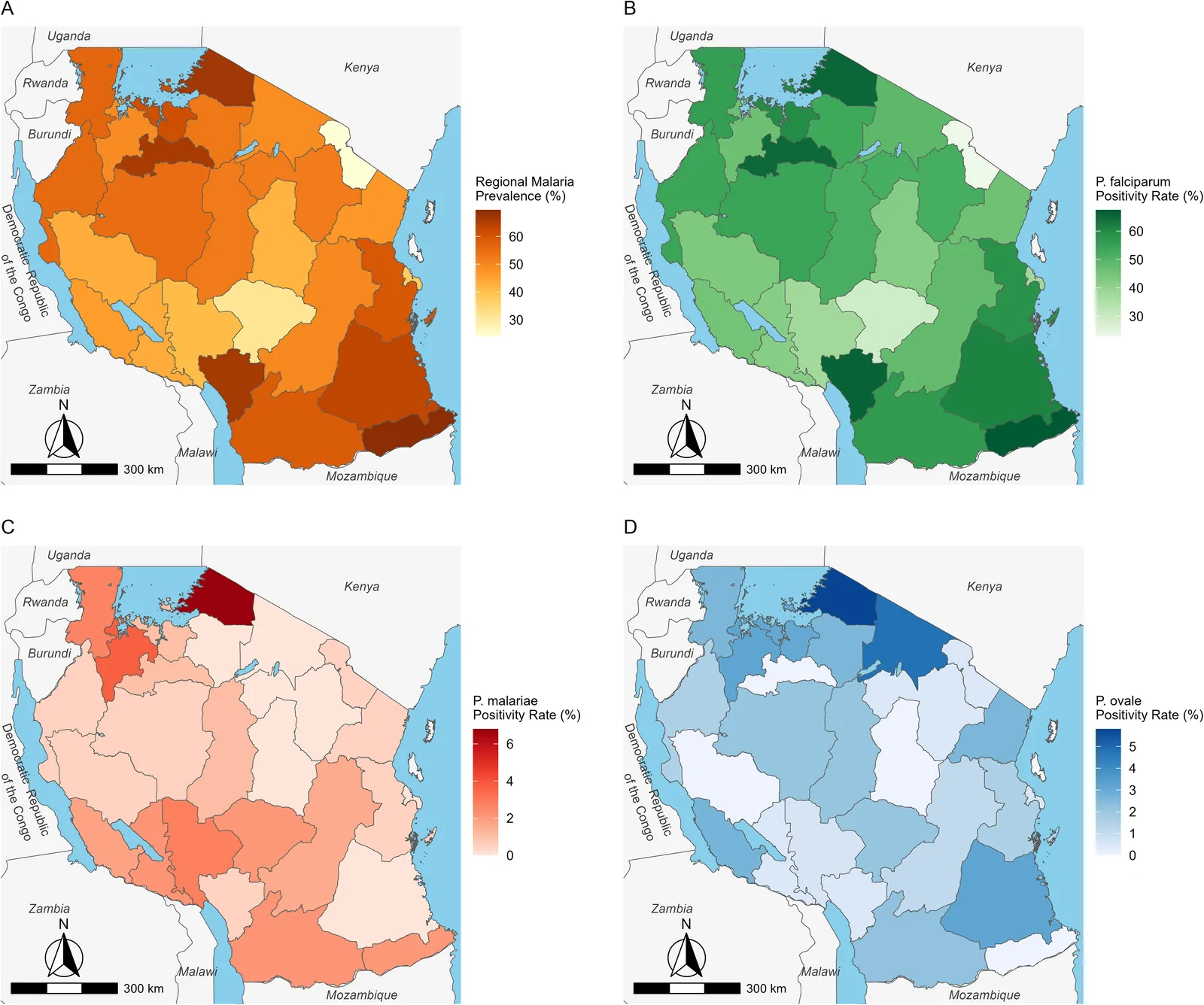
Maps showing the spatial distribution of the *Plasmodium* species across Tanzania. **(A)** Overall regional positivity rates of *Plasmodium* infections, **(B)** *P. falciparum* positivity rate, **(C)** *P. malariae* positivity rate, and **(D)** *P. ovale* positivity rate. Administrative boundaries were obtained from GADM (https://gadm.org/; license: https://gadm.org/license.html).

Significant variations in positivity rates were observed across strata for both *P. falciparum* and *P. malariae* (Fig 4). For *P. falciparum*, the chi-square test indicated significant overall differences (χ^2^ = 45.615, df = 3, p < 0.001). Post-hoc pairwise comparisons showed that both the high and moderate strata had significantly higher positivity rates than both the low (p < 0.001) and very low (p < 0.001) strata. In contrast, for *P. malariae*, Fisher’s exact test indicated significant differences (p = 0.004), with very low stratum exhibiting decreased positivity rates compared to the high stratum (p = 0.015). There was not enough evidence of variation among the remaining strata. The positivity rates across strata did not vary significantly for *P. ovale* spp. (χ^2^ = 6.904, df = 3, p = 0.075).

**Fig 4:**
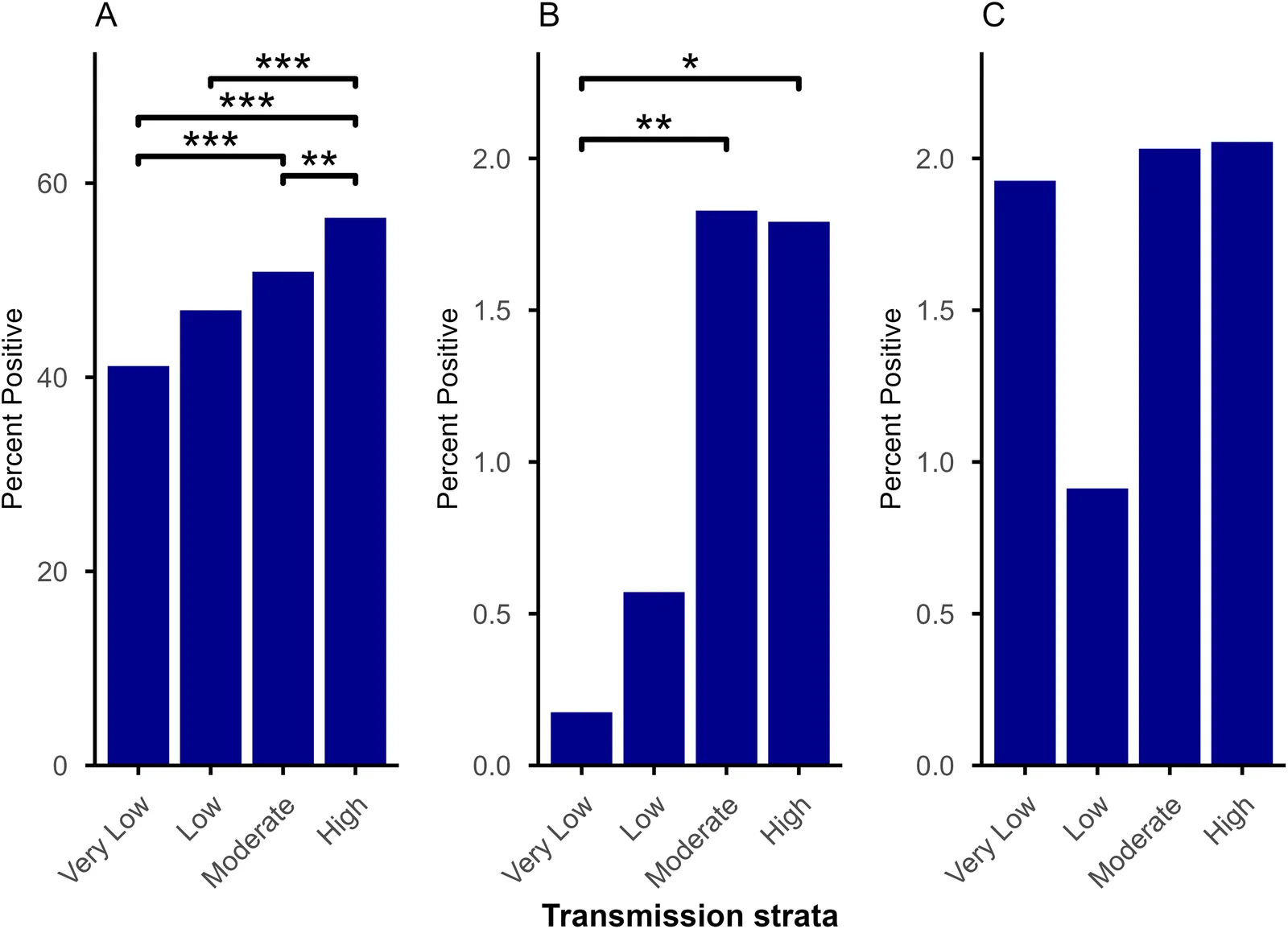
Malaria species positivity rates across transmission strata. Bars represent the percentage of samples positive for *P. falciparum*, *P. malariae*, and *P. ovale* within each transmission stratum (very low, low, moderate, and high). Pairwise comparisons of positivity rates between strata were performed using pairwise tests of proportions with Bonferroni correction. Statistically significant differences are shown as: *P < 0.05, ** P < 0.01, *** P < 0.001.

Transmission strata had a significant effect on the likelihood of *P. falciparum* infections. Tukey pairwise comparisons showed significantly higher odds of infections in the high stratum than in the low stratum (OR = 1.61 p < 0.001), while low stratum had lower odds of infections than in the moderate stratum (OR = 0.67, p < 0.001). The very low stratum had significantly lower odds than both the moderate (OR = 0.727, p < 0.001) and high strata (OR = 0.674, p < 0.001) as shown in **Table 5**. Differences between the low and moderate strata and between the low and very low strata were not statistically significant (p > 0.05). The positivity rates of *P. malariae*, were generally comparable across strata, with the exception that the very low stratum had significantly lower odds compared to the high stratum (OR = 0.237, p = 0.033). Comparisons of the odds of infections between low and moderate (p = 0.312, low and high (p = 0.157), and moderate and very low (p = 0.075) showed no strong evidence of variation. The estimated probability of *P. ovale* infection ranged from 0.8% in the low stratum to 2.0% in the high stratum. Tukey pairwise comparisons revealed no significant differences in the probabilities of infection among strata (all p > 0.05), indicating a similar likelihood of *P. ovale* infections across all strata. Mixed-species infections were also detected across transmission strata. *P. ovale* co-infections with *P. falciparum* were the most frequent, with 21 and 26 cases detected in high and moderate strata, respectively. In contrast, mixed infections of *P. falciparum* and *P. ovale* infections were uncommon in the low and very low transmission strata, with only 6 and 8 cases detected, respectively. *P. malariae* co-infections with *P. falciparum* were also common in high and moderate transmission strata (20 and 18 cases, respectively) and were rare in other strata. Triple infections involving *P. falciparum*, *P. malariae*, and *P. ovale* were rare, with two cases detected in the high stratum and four cases in the moderate stratum.

**Table 5:** Pairwise comparisons of malaria positivity rates across transmission strata.

Transmission Strata Comparison	*P. falciparum*		*P. malariae*		*P. ovale*	
	Odds ratio	Adjusted p-value	Odds ratio	Adjusted p-value	Odds ratio	Adjusted p-value
High vs Low	1.61	<0.001	2.53	0.157	2.61	0.135
High vs Moderate	1.08	0.666	1.17	0.919	0.89	0.962
High vs Very Low	1.48	<0.001	4.22	0.033	1.23	0.918
Low vs Moderate	0.67	<0.001	0.463	0.312	0.34	0.069
Low vs Very Low	0.92	0.838	1.67	0.859	0.47	0.416
Moderate vs Very Low	1.38	0.001	3.6	0.075	1.37	0.735

Pairwise comparisons were adjusted using Tukey’s method for multiple comparisons. Tests were performed on the log odds ratio scale.

### Variability by age group

The analysis of positivity rates for *Plasmodium* species across age groups revealed significant variations for *P. falciparum* and *P. malariae*, but no significant differences for *P. ovale*. For *P. falciparum*, the Chi-Square test showed a significant variation in positivity rates (χ^2^ = 33.373, df = 2, p < 0.001), with both under-fives (p < 0.001) and adults (p < 0.001) demonstrating significantly lower positivity rates than school children, as shown in Fig 5. No significant difference was observed between adults and under-fives (p = 0.95). Similarly, for *P. malariae*, age was also a significant factor (χ^2^ = 7.763, df = 2, p = 0.021), with the adults having a significantly lower positivity rate compared to the school children (p = 0.031). However, no significant differences were found in under-fives and both school children (p = 0.194) and adults (p = 1.000). For *P. ovale*, the Chi-Square test indicated no significant differences across age groups (χ^2^ = 5.424, df = 2, p = 0.066).

**Fig 5:**
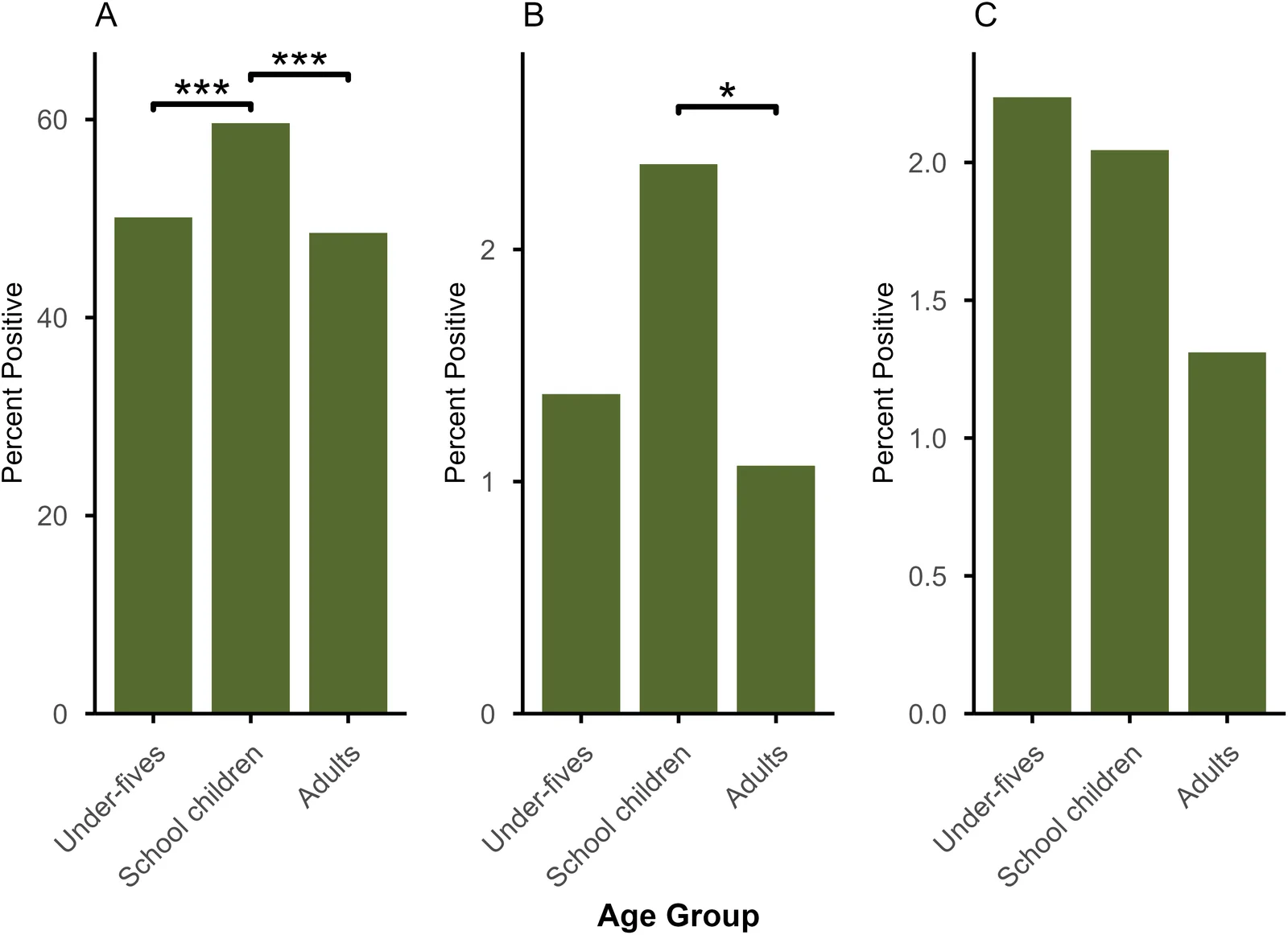
Analysis of *P. falciparum*, *P. malariae*, and *P. ovale* positivity rates across age groups. Bars represent the percentage of positive samples within each age category. Pairwise comparisons of positivity rates between groups were performed using pairwise tests of proportions with Bonferroni correction. Statistically significant differences are shown as: *P < 0.05, **P < 0.01, ***P < 0.001.

Age significantly influenced the likelihood of *P. falciparum* and *P. malariae* infection. For *P. falciparum,* school children had higher odds of infection compared to adults (OR = 1.57, p < 0.001) and under-fives (OR = 1.47, p < 0.001), while no significant difference was observed between adults and under-fives (OR = 0.94, p = 0.555). For *P. malariae*, school children also had higher odds of infection compared to adults (OR = 2.25, p = 0.021), but no significant differences were found between under-fives and adults (OR = 0.77, p = 0.626) or school children (OR = 0.58, p = 0.118). For *P. ovale*, adults showed a nearly significant lower likelihood of infection compared to under-fives (OR = 0.58, p = 0.059) and a non-significant lower likelihood compared to school children (OR = 0.64, p = 0.292). The odds of infections were similar between under-fives and school children (OR = 1.10, p = 0.939) as shown in Table 6.

**Table 6:** Pairwise comparisons of malaria positivity rates by age groups.

Age Group Comparison	*P. falciparum*		*P. malariae*		*P. ovale*	
	Odds ratio	Adjusted p-value	Odds ratio	Adjusted p-value	Odds ratio	Adjusted p-value
Adults vs Under-fives	0.94	0.556	0.77	0.627	0.58	0.059
Adults vs School children	0.64	<0.001	0.45	0.021	0.64	0.292
Under-fives vs School children	0.68	<0.001	0.58	0.118	1.09	0.939

Pairwise comparisons were adjusted using Tukey’s method for multiple comparisons. Tests were performed on the log odds ratio scale.

## Discussion

Malaria control efforts in Tanzania remain heavily focused on *P. falciparum*; however, this falciparum-centric approach may overlook the contribution of other *Plasmodium* species to the overall disease burden. Non-falciparum species, such as *P. malariae* and *P. ovale* spp., which are often present at low densities and co-infect with *P. falciparum*, can evade routine diagnostics and remain underreported. This study employed highly sensitive molecular techniques to investigate their distribution across Mainland Tanzania to provide the first report of the different *Plasmodium* species in all regions. The findings revealed that, although less prevalent, non-falciparum species are more widespread than previously recognized and may play a significant yet silent role in sustaining malaria transmission. These findings support the necessity to broaden malaria surveillance and management strategies beyond *P. falciparum*.

The findings reaffirm that *P. falciparum* is the predominant species in Mainland Tanzania, consistent with long-standing patterns observed in SSA [50]. However, the detection of *P. malariae* and *P. ovale* spp., including their occurrence as mono-infections, reveals a more complex species landscape than is often recognized. These non-falciparum species were traditionally considered rare and clinically insignificant, yet their presence, even at lower prevalence, suggests they may silently contribute to malaria morbidity [51]. Their tendency to co-infect with *P. falciparum* at low densities may allow them to evade routine diagnostic methods, further complicating surveillance and treatment efforts [52,53]. Moreover, current treatment algorithms in Tanzania are primarily designed for *P. falciparum*. Although artemisinin-based combination therapies are effective against blood-stage infections of non-falciparum species, existing approaches may not fully address the biological characteristics and radical cure requirements of *P. ovale*. This raises concerns about the adequacy of existing case management strategies for these infections caused by non-falciparum species.

Although detected at lower abundances than *P. falciparum*, both *P. malariae* and *P. ovale* spp. were found to be more widely distributed than previously recognised. This broader distribution may indicate either a true increase or simply reflect previously limited data. Previous studies often concentrated on a few areas, leaving many regions under-represented [35,40]. Therefore, it is challenging to determine whether these species are newly emerging or were previously present but undetected. Interestingly, *P. malariae* and *P. ovale* spp. were not restricted to high malaria transmission areas, suggesting that these species may persist even in areas with declining malaria transmission. Moreover, pairwise comparisons showed no significant differences in the probabilities of *P. ovale* infections across transmission strata, suggesting a stable prevalence independent of transmission intensity. Likewise, the odds of *P. malariae* infection did not vary significantly among strata, indicating a consistent presence across different transmission settings. These findings differ from those of a 2021 study, which reported a significant effect of transmission strata on the positivity rates of these species [40]. The discrepancy may reflect the narrower geographic scope of the previous study compared to the present analysis. These findings highlight the importance of including all *Plasmodium* species in malaria control and elimination efforts, even in areas with reduced transmission.

*P. malariae* was most frequently detected in regions of moderate to high *P. falciparum* transmission; however, its presence was not uniform, being absent in several regions, including Arusha, Dodoma, Dar es Salaam, Lindi, Manyara, and Simiyu. In contrast, *P. ovale* spp. demonstrated a broader but still patchy distribution, appearing in both high and low transmission zones. This raises questions about their potential role in sustaining low-level malaria transmission, particularly as control interventions intensify [54,55]. Their occurrence in areas with various transmission intensities suggests that their persistence is not solely dependent on high transmission settings. This supports growing evidence that these species may occupy ecological or host niches that enable them to endure even under changing malaria transmission dynamics [56]. These findings call for malaria policies to expand current surveillance systems and adapt intervention strategies to target all human malaria species circulating in the country.

The elevated presence of *P. malariae* in Mara and *P. ovale* spp. in both Mara and Arusha regions underscores the need for localized surveillance, as species distribution varies regionally. Mara region continues to show unusually high levels of non-falciparum malaria species, consistent with findings from a similar study conducted in 2021 [40]. Although overall prevalence has declined compared to the findings of 2021’s study, it remains significantly higher than in other regions, raising important questions [40]. Given that both studies conducted so far have been cross-sectional, it's difficult to capture the true dynamics of these non-falciparum infections in this area. What’s particularly puzzling is that neighboring regions report much lower rates, making it less likely that these cases are solely attributable to spillovers from the nearby country, Kenya. Apart from Mara, regions like Geita and Kagera stand out for their relatively high prevalence of non-falciparum species, highlighting possible ecological or host-related factors that support their persistence and positioning these areas as potential hotspots for these under-reported parasites. This calls for further research and closer monitoring, as existing interventions may not be adequate to address the unique transmission patterns in these regions.

The present study found that school children bear the greatest burden and the highest odds of *P. malariae* infection. The elevated positivity rates and infections in this group likely reflect a combination of exposure patterns, parasite biology, and immunological factors. *P. malariae* can persist at low densities without symptoms, allowing chronic infections to accumulate over time in this age group [57,58]. This is because school children are old enough to experience fewer symptoms yet not fully immune, allowing infections to persist at subclinical, low-density levels [59]. Moreover, school children are more mobile and frequently exposed to outdoor environments, especially during dusk, which increases their risk of mosquito bites [57]. While younger children may benefit from targeted interventions, school children often do not equally benefit from malaria control interventions, making them a vulnerable reservoir for ongoing transmission [58,59]. Interestingly, school children often carry a significant burden of asymptomatic cases; this study reveals a different pattern, suggesting a higher frequency of symptomatic cases in this age group. While it is challenging to confirm a clear shift in malaria burden from under-fives to school children, these results indicate that infections in this group may increasingly lead to clinical symptoms. These findings highlight the need to extend malaria interventions to school children to reduce their burden and curb transmission.

The observed high GMPDs among under-fives in this study are consistent with previous reports describing higher mean *P. falciparum* parasite densities in younger age groups [49,60,61]. The significantly elevated densities in under-fives and school children suggest that these groups contribute to ongoing transmission and remain transmission [62]. Although gametocyte carriage was not assessed in the present study, higher parasite densities have previously been associated with increased gametocyte densities, the transmissible stage of the parasite [63]. Therefore, the higher parasite densities observed in these age groups may indicate a greater potential for sustaining malaria transmission. However, further studies incorporating measurements of gametocyte or mosquito infectivity are needed to determine their role as transmission reservoirs. These findings nevertheless highlight under-fives and school-aged children as important target populations for malaria surveillance and control interventions. Similar age-related density patterns have been observed in previous studies conducted elsewhere, reinforcing the role of young children as key contributors to malaria transmission [64,65]. The modest yet significant age effect for *P. ovale* spp. infections could be due to the gradual acquisition of immunity. These findings parallel reports from previous studies, where younger children exhibited higher densities than older individuals with more developed immunity [60,61]. In contrast, age had no significant effect on *P. malariae* parasite densities, which may reflect its ability to persist at low densities due to its slow intraerythrocytic replication cycle, lower immunogenicity, and potential for long-term chronic infections that elicit minimal inflammatory response across age groups [66,67]. Collectively, these findings emphasize the need for age-targeted approaches in intervention programs.

A key strength of this study is its national scope; it is the first to investigate *Plasmodium* species positivity rates across all 26 regions of Mainland Tanzania using molecular diagnostic techniques, offering a comprehensive and representative assessment of species distribution. Nonetheless, several limitations should be considered. First, only a subset of collected samples was analysed, which, despite being randomly selected, may limit the precision of the estimates of positivity rates, particularly for less common species. Second, the cross-sectional design captures data at a single point in time, limiting the ability to detect seasonal trends or infection dynamics. The present survey was conducted between January and August 2023 and therefore may not fully capture seasonal fluctuations in species distribution. Previous studies from coastal Tanzania have demonstrated temporal variation in non-falciparum species, particularly *P. ovale*, whose prevalence may fluctuate independently of *P. falciparum* and may persist during periods of lower falciparum transmission. Furthermore, longitudinal studies from Tanzania have shown sustained transmission of *P. malariae* and *P. ovale* despite substantial reductions in *P. falciparum* prevalence, suggesting distinct ecological and epidemiological dynamics of non-falciparum species [68]. Consequently, the species distributions reported here should be interpreted within the context of the survey period and may not fully represent year-round patterns. These temporal aspects are important in malaria epidemiology, particularly for non-falciparum species that may follow different patterns of persistence and recrudescence. Another limitation of this study is that *P. vivax* was not included in the molecular analysis; although *P. vivax* is considered rare in the country because it was detected in few samples in 2021 [40] and in other studies [69,70], its current presence could not be assessed. Lastly, being a health facility-based study, study may have missed asymptomatic non-falciparum infections, which often occur at low densities and do not prompt care-seeking, potentially underestimating their true prevalence. Further research using longitudinal approaches is needed to further understand the epidemiology of different *Plasmodium* species.

## Conclusion

The study found high positivity rates and dominance of *P. falciparum* the widespread occurrence of *P. malariae* and *P. ovale* spp. across multiple regions of Tanzania, indicating a broader distribution than previously reported. Although both species were found at lower abundance compared to *P. falciparum*, they were present across a range of transmission settings, including areas with declining malaria prevalence. No significant differences were observed in the odds of infections for *P. malariae* or *P. ovale* spp. across transmission strata, suggesting a consistent presence irrespective of transmission intensity. A disproportionate burden of *P. malariae* infections was observed among school children, with higher positivity rates observed in Geita, Kagera, and Mara regions. These findings underpin the call to expand current surveillance systems and adapt intervention strategies to target all human malaria species and all age groups, especially those historically overlooked in control efforts.
